# High expression of COPZ2 is associated with poor prognosis and cancer progression in glioma

**DOI:** 10.3389/fnmol.2024.1438135

**Published:** 2024-07-31

**Authors:** Zhi Geng, Chunyan Mu, Yuxiang Qiu, Yuchen Tang, Mingyu Su, Chuanxi Tang, Lei Zhang

**Affiliations:** ^1^Department of Pediatric Surgery, Tengzhou Central People's Hospital, Tengzhou, Shandong, China; ^2^Department of Neurobiology, Xuzhou Key Laboratory of Neurobiology, Xuzhou Medical University, Xuzhou, Jiangsu, China; ^3^Department of Neurology, Affiliated Hospital of Xuzhou Medical University, Xuzhou, Jiangsu, China

**Keywords:** glioma, COPZ2, prognosis, migration, proliferation

## Abstract

**Background:**

Coatomer protein complex zeta 2 (COPZ2) is a member of heptameric coatomer protein complex I and has been reported to be involved in various tumors. However, COPZ2’s potential involvement in glioma remains to be explored.

**Methods:**

The COPZ2 expression and related clinical data were obtained from The Cancer Genome Atlas (TCGA). TIMER2.0 and the Ualcan database were utilized to assess the COPZ2 expression in various tumors. Univariable, multivariate Cox regression, Kaplan–Meier methods, nomogram analysis, and ROC curve analysis were carried out to assess the relationship of COPZ2 and other prognostic factors with glioma. The LinkedOmics database was used to predict the potential biological mechanism of COPZ2 in glioma. We also conducted *in vitro* experiments to evaluate the functional role and mechanism of COPZ2 in glioma cell lines.

**Results:**

We found that COPZ2 was highly expressed in glioma and it was associated with age and WHO grades. Kaplan–Meier survival curves, Cox analysis, nomogram analysis, and ROC curve showed that COPZ2 was a disadvantageous factor in poor glioma prognosis. The functions of COPZ2 and co-expression genes were significantly associated with neutrophil-mediated immunity, granulocyte activation, and response to interferon-gamma. In addition, COPZ2 knockdown significantly inhibited the proliferation, migration, and invasion of glioblastoma cells. Mechanistically, COPZ2 suppressed tumor development by participating in the regulation of the PI3K-AKT signaling pathway.

**Conclusion:**

Our results demonstrated that the elevation of COPZ2 was associated with the prognosis and progression of glioma, and it might be a potential diagnostic and prognostic biomarker for glioma.

## Introduction

Glioma is the most prevalent primary brain tumor, accounting for 81% of central nervous system malignancies (Takacs et al., 2021). These tumors typically originate in glial or pre-glial cells and can progress into astrocytoma, oligodendroglioma, and other forms. The World Health Organization classifies glioma into four grades, with grades 1 and 2 referred to as Low-grade gliomas (LGG) and grades 3 and 4 as High-grade gliomas (HGG) (Louis et al., 2021). Higher grades are generally associated with a poorer prognosis. The 10-year survival rate for low-grade gliomas is 47%, with a median survival time of 11.6 years. In contrast, patients with grade 3 glioma have a median overall survival of approximately 3 years, while those with grade 4 glioma experience an even worse median overall survival of just 15 months. Glioblastoma is the most common form of grade 4 glioma (Kong et al., 2018). The traditional treatment approaches for glioma include surgical resection, temozolomide chemotherapy, and radiotherapy. However, complete resection of glioma is challenging and has a poor prognosis (Davis, 2016). Temozolomide is currently a standard chemotherapy drug used for clinical glioma treatment. It induces tumor cell death by promoting the alkylation of glioma at multiple gene sites (Tomar et al., 2021). However, clinical observations have shown that glioma recurrence still poses a risk after temozolomide treatment. Therefore, there is an urgent need for more effective molecular-targeted therapies.

A growing body of research has highlighted the role of various cellular pathways and molecular mechanisms in glioma progression. One such mechanism involves the coatomer protein complex subunit zeta-2 (COPZ2). COPZ2 is a member of heptameric coatomer protein complex I (COPI), which is mainly involved in intracellular vesicle trafficking, particularly within the Golgi apparatus and between the Golgi and the endoplasmic reticulum (ER). This protein plays a critical role in the maintenance of Golgi structure and function, which is essential for proper cellular trafficking and protein sorting (Futatsumori et al., 2000). In addition to this, COPZ2 is also responsible for the assembly of coated vesicles on Golgi membranes, autophagy, lipid homeostasis, and endosome maturation (Shtutman et al., 2011; Di Marco et al., 2020). Many studies reported that COPZ2 was differentially expressed in a variety of tumors, such as thyroid cancer, bladder cancer, and hepatocellular carcinoma (Tsuruta et al., 2011; Huang et al., 2023). In addition, high expression of COPZ2 was associated with an unfavorable prognosis in cancer patients (Zhang et al., 2022). A previous study showed that overexpression of COPZ2 protected tumor cells from killing by COPZ1 knockdown (Shtutman et al., 2011). Furthermore, COPZ2 has been implicated in the regulation of autophagy (Shtutman et al., 2011), a cellular degradation process that is often hijacked by cancer cells to support their metabolic needs and resist apoptosis. In the context of glioma, it has been suggested that COPZ2 may have a significant pathophysiological role. Aberrant expression of COPZ2 has been detected in glioma tissues compared to normal brain tissues, indicating its potential involvement in tumorigenesis. The dysregulation of COPZ2 can disrupt normal vesicular transport and protein processing, leading to altered cell signaling pathways that promote tumor growth and survival. However, no studies focused on the relationship between COPZ2 expression and glioma, and its clinical significance, biological roles, and underlying molecular mechanisms for glioma are still unclear.

Given its central role in vesicular trafficking and its emerging importance in glioma pathology, COPZ2 represents a promising target for therapeutic intervention. In this research, we elucidate the crucial role of COPZ2 in glioma through a combination of bioinformatics analysis and experimental investigations. Additionally, it provides preliminary insights into the molecular mechanisms through microarray analysis. Overall, our study suggests that COPZ2 is a promising diagnostic and prognostic target for glioma patients.

## Methods

### Data acquisition and preprocessing

The RNA-seq data for the STAR process of TCGA-GBMLGG (*n* = 706) were obtained from The Cancer Genome Atlas (TCGA) database.^1^ The TCGA dataset consists of gene expression data and clinical data. Clinical data included gender, age, race, WHO grade, IDH1 status, 1p/19q status, survival status, and survival time. The gene expression data were then converted from FPKM format to TPM format. The differences of various clinicopathological parameters were compared between the high-COPZ2 and low-COPZ2 expression groups. A Kaplan–Meier survival analysis was conducted using the expression levels of COPZ2 and the survival status of patients with TCGA-GBMLGG. Kaplan–Meier survival and Cox regression analyses were conducted to assess the correlation between COPZ2 expression level and prognosis. TIMER 2.0^2^ was used to analyze the pan-cancer expression of COPZ2 (Li et al., 2020). The Ualcan database^3^ was used to analyze the expression of COPZ2 in glioma (Chandrashekar et al., 2022).

### Construction of prognostic model

Nomogram was constructed to predict the OS rates at 1 year, 2 years, and 3 years by using the RMS package and survival package (Balachandran et al., 2015). The calibration of the nomogram was performed to visualize predicted probabilities. Time-dependent receiver operating characteristic (ROC) curves were constructed to assess the diagnostic value of COPZ2 for glioma by using the timeROC package.

### LinkedOmics database

The LinkedOmics database^4^ is a comprehensive analysis platform, it contains 32 TCGA Cancer types and 10 CPTAC cancer cohorts (Vasaikar et al., 2018). The “LinkFinder” module was used to perform COPZ2 gene co-expression analysis in the TCGA-GBMLGG dataset based on the Spearmen correlation coefficient. The “LinkInterpreter” module was used to conduct Gene Oncology (GO) and Kyoto Encyclopedia of Gene and Genomes (KEGG) pathways analysis. The rank criterion was an FDR < 0.05.

### Protein–protein interaction network

The protein interaction networks among genes enriched in each item were retrieved and downloaded from STRING^5^ (Szklarczyk et al., 2021), selecting the interaction score > 0.4 in the PPI network.

### Immunohistochemistry

The tissue microarray comes from our previously published article (Tang et al., 2020). The slides were blocked with 5% normal goat serum and incubated with anti-COPZ2 (1, 500, Proteintech) at 4°C. After washing with phosphate-buffered saline, the slides were incubated with goat anti-rabbit horseradish peroxidase (Vector Laboratories, Burlingame, CA) for 30 min at room temperature. A DAB kit (MXB Biotechnologies, DAB-1031, China) was used to detect the immunohistochemical reactions. The photograph was obtained under a phase contrast light microscope (Olympus).

### Cell culture and transfection

Human glioma cell lines U251 and U87 cell lines were obtained from the China Center for Type Culture Collection (CCTCC, China). The cells were cultured in DMEM with 10% fetal bovine serum, 100 U/mL penicillin, and 100 μg/mL streptomycin at 37°C with 5% CO2. For the rescue experiments, cells were incubated with 5 μg/mL SC79 (Zhou et al., 2018) (Macklin, China). When the confluence reached 80%, the cells were transfected with COPZ2 small interfering RNA (siRNA) or negative control (NC) siRNA by using Lipofectamine 3,000 based on the manufacturer’s instructions. The siRNA sequences were human COPZ2-1: CCAAGUAUUAUGAUGACACAU; human COPZ2-2: GCAAGUGAUCCAGAAGGUGAA.

### Cell proliferation assay

The glioma cells were plated in 96-well plates at the density of 3,000 cells/well. After the desired incubation period, the CCK-8 solution from the kit (Dojindo Laboratories, Kumamoto, Japan) is added to each well at a final concentration of 10% (10 μL per well), and the plates are incubated for an additional 1–4 h at 37°C. The solution contains a water-soluble tetrazolium salt that is reduced by dehydrogenases in the cells to form a colored formazan dye, which is soluble in the culture medium. The absorbance at 450 nm, which correlates with the number of viable cells, is then measured using a microplate reader (Synergy H1, BIOTEK, United States).

### Wound healing assay

The glioma cells were plated in 6-well plates at the density of 30,000 cells/well. When the density of cells reached 90–100% confluence, a 200-μL pipette tip was used to scratch a straight wound. A wound healing photograph was obtained after 24 h by using a microscope. The formula for calculating healing percent was as follows: healing percent = ((gap width at 0 h) − (gap width at 24 h)) / (gap width at 0 h).

### Transwell assay

The glioma cells were plated in the upper compartment of 24-well plates that had been pre-coated with an 8 μm matrix gel at the density of 3,000 cells/well, and DMEM containing 10% FBS was added to the lower compartment. After 24 h, cells on the top surface of the membrane were wiped off, and cells on the lower surface of the membrane were fixed with 4% paraformaldehyde and stained with 0.1% crystal violet. The photographs were acquired using a microscope.

### Quantitative reverse transcriptase-polymerase chain reaction

After 72 h of transfection, the total RNA of cells was extracted using the TRIzol reagent (Invitrogen, Carlsbad, CA, United States). Then, RNA was reversely transcribed into cDNA with the HiScript III 1st Strand cDNA Synthesis Kit (Vazyme, China). The qRT-PCR was performed by AceQ qPCR SYBR Green Master Mix (Vazyme, China) with specific primers and the Roche LightCycler 480 PCR System. The relative expression of the gene was normalized to GAPDH using the 2 − ΔΔCt method. The primer sequences used are as follows: COPZ2 forward: 5′- ATTGTGGATGGCGGTGTGATT-3′, COPZ2 reverse: 5′- TCCTTGGCAGACTGAAGAACC-3′; GAPDH forward: 5’-CTGGGCTACACTGAGCACC-3′, reverse: 5’-AAGTGGTCGTTGAGGGCAATG-3′.

### Western blot

After 72 h of transfection, proteins were extracted from the U87 and U251 cell lines using RIPA lysis buffer. The protein concentration was determined with a BCA determination kit. The proteins were separated by SDS-polyacrylamide gel electrophoresis (SDS-PAGE) and then transferred to nitrocellulose membranes (EMD Millipore, Billerica, MA). The membranes were incubated with primary antibodies (β-actin, 1: 5,000, Proteintech; COPZ2, 1:1,000, GeneTex) at 4°C overnight. Subsequently, the membranes were incubated with goat anti-rabbit secondary antibodies (1:1,000; LI-COR Biosciences). The membranes were scanned using the Odyssey Infrared Laser Scanning Image System (LI-COR Biosciences).

### Microarray assay

After 72 h of transfection, the total RNA of cells was extracted using the TRIzol reagent, and the NanoDrop 2000 and Agilent 2,100 Bioanalyzer (Aglient) were used to evaluate the total RNA quality. Then, Cy3 labeled cDNAs were synthesized and hybridized onto a human mRNA Array V4.0 (Agilent, Santa Clara, CA). The microarray Assay was performed by CapitaBio Technology. Significant differentially expressed mRNA were identified as those with |log_2_ (fold change) | > 1 and padj <0.05.

### Statistical analysis

Statistical analysis was performed using GraphPad Prism8 and R (version 4.2.1). The expression of COPZ2 between different groups has been performed by Wilcoxon rank-sum tests. Univariate and multivariate Cox regression analyses were performed to calculate the association between COPZ2 expression and clinicopathological characteristics. The survival curve was constructed using the KM method and the log-rank test. Comparisons among groups were analyzed using Student’s *t*-tests or one-way ANOVA followed by a Tukey’s post-test. Data are presented as mean ± SEM, and a *p*-value <0.05 was considered statistically significant.

## Result

### The expression of COPZ2 is upregulated in glioma

**Figure 1:**
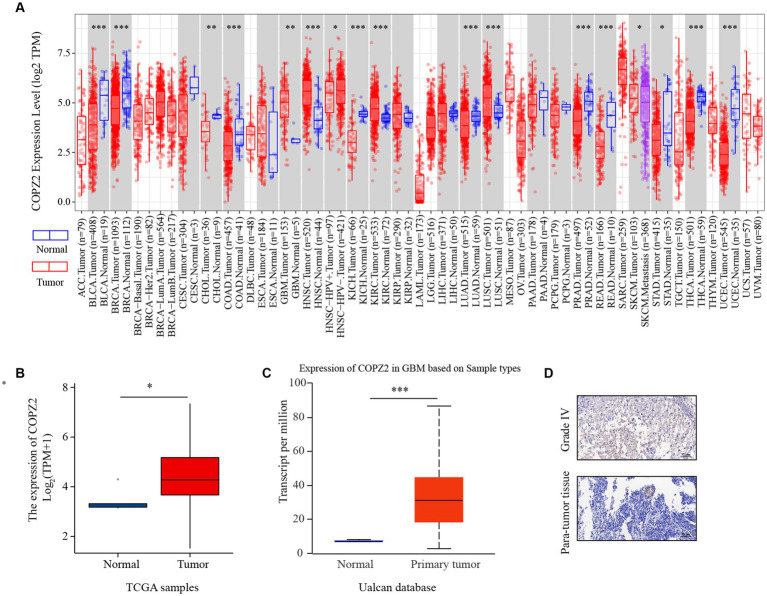
The expression of COPZ2 is upregulated in glioma. **(A)** The expression level of COPZ2 in different types of tumor tissues (red) and normal tissues (blue) was assessed in TIMER2.0 database. The level of COPZ2 is upregulated in glioma tissues compared with normal tissues in **(B)** TCGA-GBMLGG and **(C)** Ualcan database. **(D)** Representative images of COPZ2 IHC staining in glioma tissue and para-tumor tissue. Scale bars, 50 μm.

To explore the discrepancies of COPZ2 expression in human cancers, we compared the differential expression of COPZ2 in various tumor tissues and normal tissues using the TIMER 2.0 database. The results revealed that COPZ2 expression was significantly upregulated in GBM (glioblastoma IV grade), HNSC (Head and Neck squamous cell carcinoma), KIRC (Kidney renal clear cell carcinoma), LUSC (Lung squamous cell carcinoma). Nevertheless, COPZ2 expression was significantly reduced in BLCA (bladder urothelial carcinoma), BRCA (breast invasive carcinoma), CHOL (cholangiocarcinoma), KICH (kidney chromophobe), LUAD (lung adenocarcinoma), PRAD (prostate adenocarcinoma), READ (Rectum adenocarcinoma), STAD (stomach adenocarcinoma), THCA (Thyroid carcinoma), and UCEC (uterine corpus endometrial carcinoma) than in normal controls (Figure 1A). In addition, we verified that COPZ2 expression levels were upregulated in glioma with TCGA, and Ualcan Databases (Figures 1B,C). The IHC also showed that COPZ2 protein expression was higher in glioma tissues (Figure 1D). The above data demonstrated that COPZ2 was increased in glioma tissue.

### Relationship of COPZ2 expression with clinical parameters of patients with glioma

**Figure 2:**
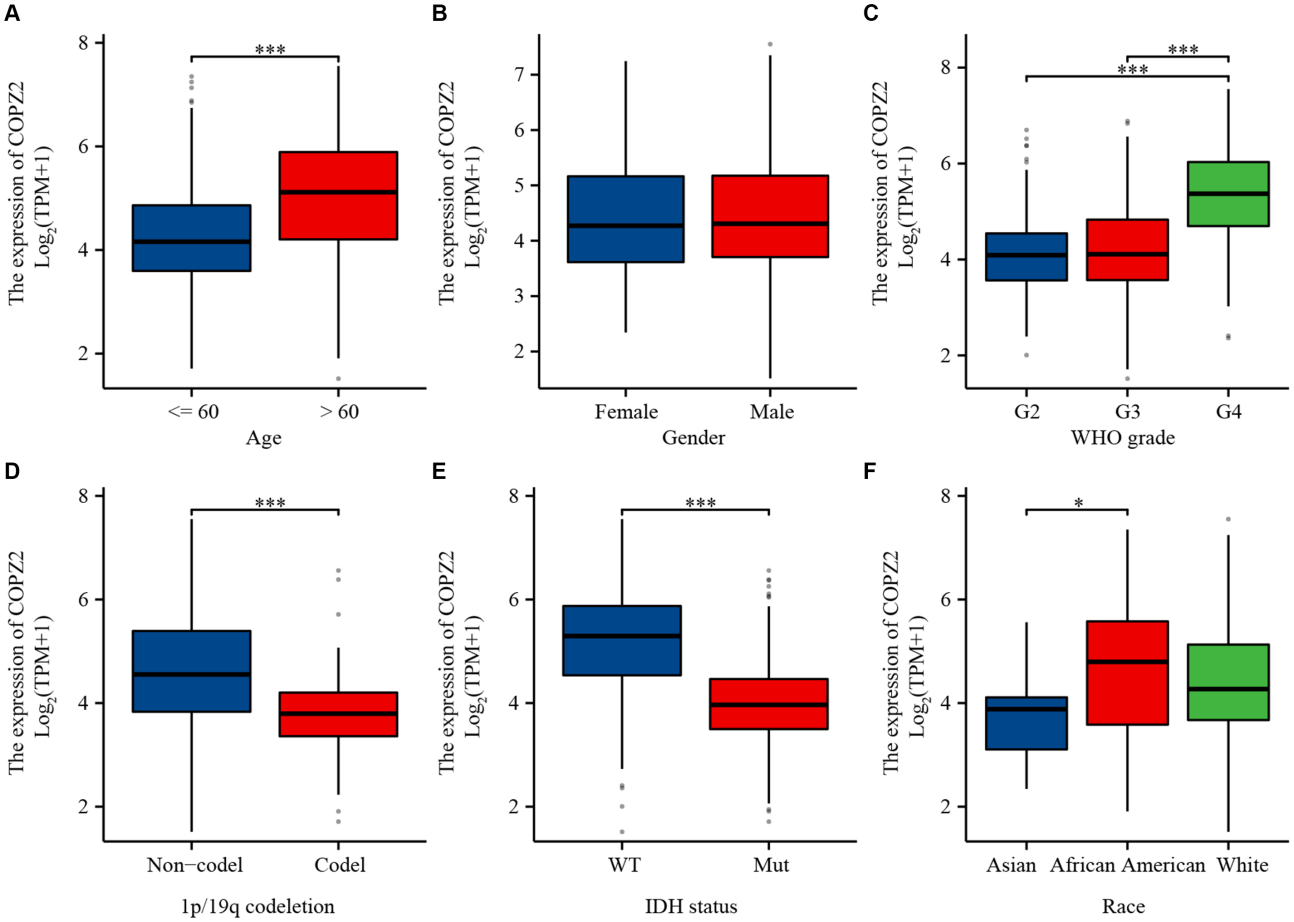
The relationship between COPZ2 expression and clinical characteristics. **(A–F)** Expression level of COPZ2 in patients with different ages, gender, WHO stages, 1p/19q codeletion, IDH status, and race. *p < 0.05, **p < 0.01, ***p < 0.001.

To investigate the relationship between COPZ2 expression and clinicopathological characteristics in glioma, the clinical characteristics of 699 cases were obtained from the TCGA database. Then, the patients were stratified into “high COPZ2” and “low COPZ2” groups according to the median COPZ2 level (Table 1). As shown in Figure 2, higher COPZ2 expression was observed in aged >60 patients, WHO grades 3 and 4, and African American. Secondly, COPZ2 was more expressed in the IDH wild-type and non-codeletion of 1p/19q group.

**Table 1:** Clinicopathologic features of low and high COPZ2 expression groups in glioma.

Characteristics	Low expression of COPZ2	High expression of COPZ2	*p* value
*n*	349	350	
Age, *n* (%)			<0.001
<=60	311 (44.5%)	245 (35.1%)	
>60	38 (5.4%)	105 (15%)	
WHO grade, *n* (%)			<0.001
G2	141 (22.1%)	83 (13%)	
G3	139 (21.8%)	106 (16.6%)	
G4	26 (4.1%)	142 (22.3%)	
IDH status, *n* (%)			<0.001
WT	49 (7.1%)	197 (28.6%)	
Mut	297 (43.1%)	146 (21.2%)	
1p/19q codeletion, *n* (%)			<0.001
Non-codel	214 (30.9%)	306 (44.2%)	
Codel	133 (19.2%)	39 (5.6%)	
Gender, *n* (%)			0.853
Female	150 (21.5%)	148 (21.2%)	
Male	199 (28.5%)	202 (28.9%)	
Race, *n* (%)			0.031
Asian	11 (1.6%)	2 (0.3%)	
African American	14 (2%)	19 (2.8%)	
White	321 (46.8%)	319 (46.5%)	
Histological type, *n* (%)			<0.001
Astrocytoma	99 (14.2%)	97 (13.9%)	
Oligoastrocytoma	77 (11%)	58 (8.3%)	
Oligodendroglioma	147 (21%)	53 (7.6%)	
Glioblastoma	26 (3.7%)	142 (20.3%)	

### COPZ2 is associated with the prognosis of glioma patients

**Figure 3:**
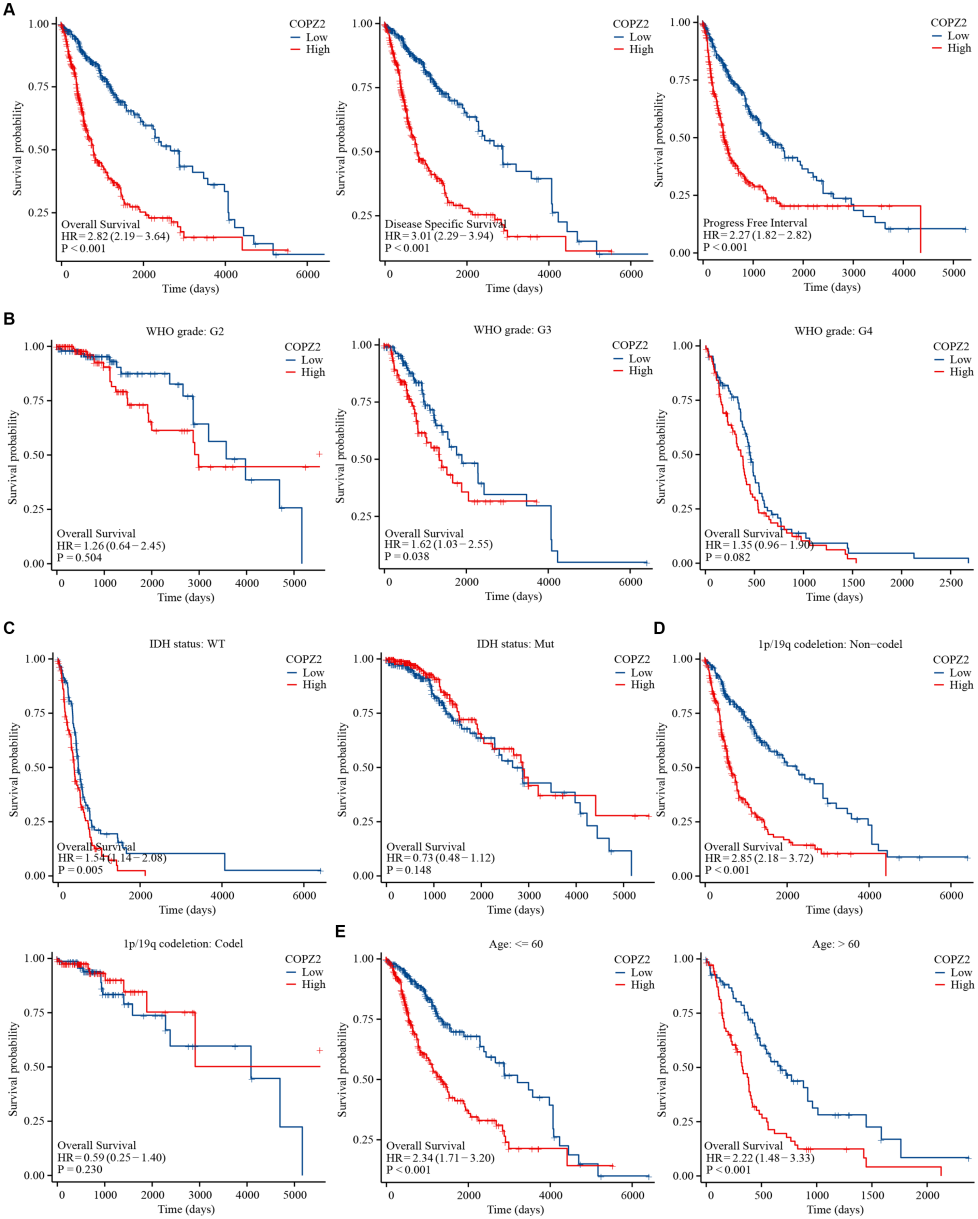
High level of COPZ2 was associated with poor prognosis. **(A)** High COPZ2 was correlated with poor prognosis of glioma in OS, DSS, and PFI. **(B)** Subgroup survival analyses for correlations between COPZ2 expression and WHO grade. **(C)** Subgroup survival analyses for correlations between COPZ2 expression and IDH status. **(D)** Subgroup survival analyses for correlations between COPZ2 expression and 1p/19q codeletion status. **(E)** Subgroup survival analyses for correlations between COPZ2 expression and age. OS, overall survival; DSS, disease-specific survival, PFI, progress free interval.

**Figure 4:**
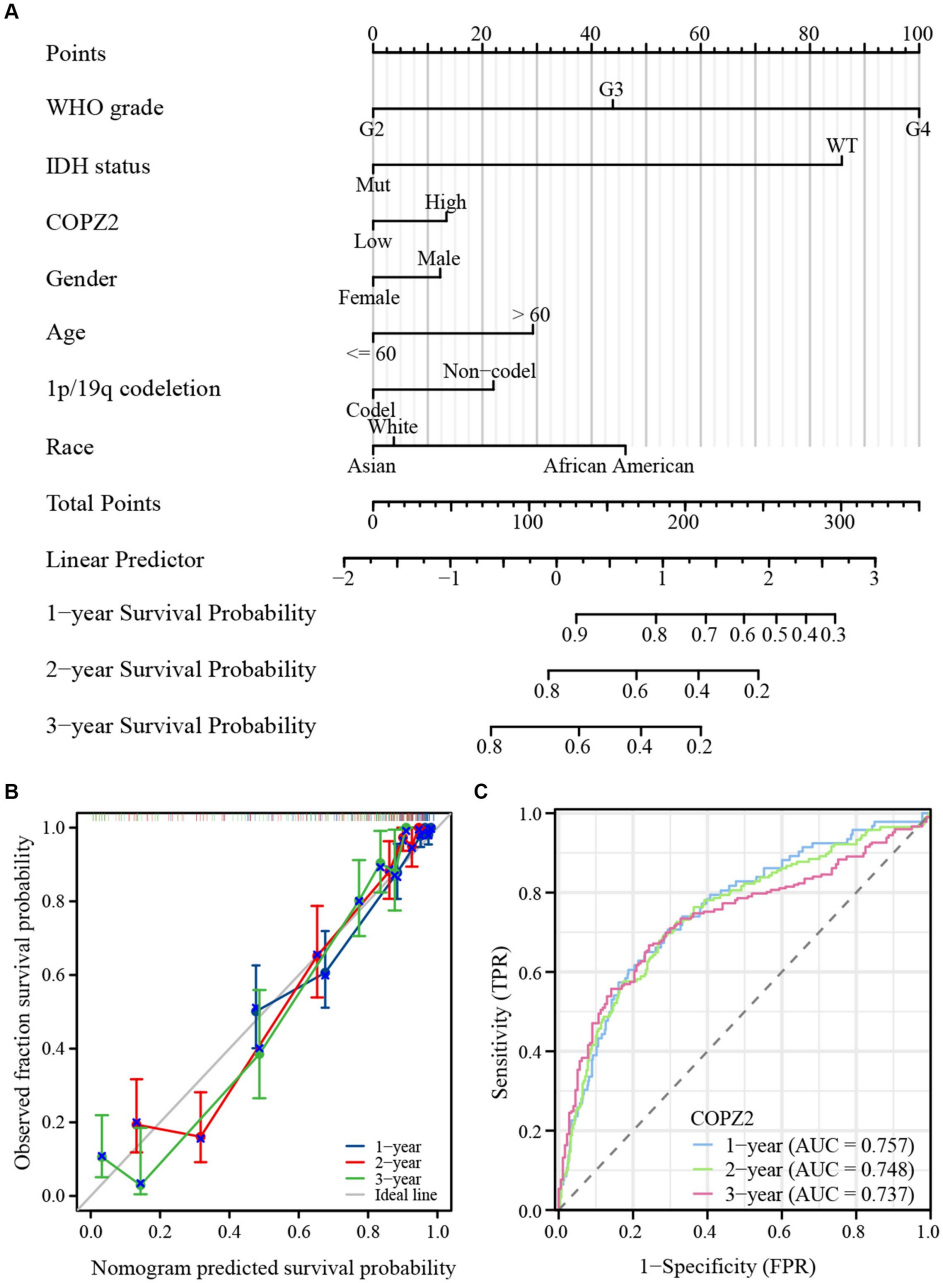
Diagnostic value of COPZ2 in glioma. **(A)** A nomogram was established based on the multivariate survival analysis. **(B)** Calibration plots of the nomogram. **(C)** Time-dependent ROC curves.

To explore the correlation between COPZ2 and the prognosis in glioma, Kaplan–Meier analysis showed that patients with high expression of COPZ2 exhibited poor overall survival (OS) (Hazard Ratio (HR) = 2.82, *p* < 0.001), disease-specific survival (DSS) (HR = 3.01, *p* < 0.001), and progress free interval (PFI) (*p* = 2.27, *p* < 0.001) in TCGA database (Figure 3A). Subgroup analysis of OS based on age, WHO grade, IDH1 status, and 1p/19q status also showed that poor prognosis was accompanied by increased COPZ2 level in the WHO grade 3, IDH wild-type, non-codeletion of 1p/19q, aged >60, and aged ≤60 group (Figures 3B–E) and the expression of COPZ2 was not significantly related with prognosis in WHO grade 4, IDH Mutant and codeletion of 1p/19q. We further conducted univariate and multivariate Cox regression analyses to identify risk factors (Table 2). In univariate Cox regression analysis, the age, WHO grade, 1p/19q codeletion, IDH status, and COPZ2 expression were correlated with OS in glioma patients. The high COPZ2 expression was associated with a worse prognosis in glioma (HR = 2.824, *p* < 0.001). However, the results of the multivariate analysis showed no statistical significance. Nonetheless, the age, WHO grade, and IDH status also were correlated with OS in multivariate Cox regression analysis (Table 2). Based on univariate and multivariate Cox regression models, we constructed nomograms predicting the 1, 2, and 3-year survival rates of glioma patients (Figure 4A). The nomogram-predicted survival probability calibration plots were close to the ideal (Figure 4B), and time-dependent ROC analysis showed that the area under the curve (AUC) in predicting OS at 1, 2, and 3 years were 0.757, 0.748, and 0.737, respectively (Figure 4C). These data suggested that COPZ2 might be a biomarker indicating a poor prognosis of glioma.

**Table 2:** Univariate and multivariate Cox regression analyses of the overall survival of glioma.

Characteristics	Total (*N*)	Univariate analysis		Multivariate analysis	
		Hazard ratio (95% CI)	*p* value	Hazard ratio (95% CI)	*p* value
Age	698				
<=60	555	Reference		Reference	
>60	143	4.696 (3.620–6.093)	**<0.001**	1.527 (1.119–2.083)	**0.008**
WHO grade	636				
G2	223	Reference		Reference	
G3	245	2.967 (1.986–4.433)	**<0.001**	1.993 (1.300–3.055)	**0.002**
G4	168	18.600 (12.448–27.794)	**<0.001**	4.831 (2.871–8.129)	**<0.001**
IDH status	688				
WT	246	Reference		Reference	
Mut	442	0.116 (0.089–0.151)	**<0.001**	0.308 (0.207–0.459)	**<0.001**
Gender	698				
Female	297	Reference			
Male	401	1.250 (0.979–1.595)	0.073		
1p/19q codeletion	691				
Non-codel	520	Reference		Reference	
Codel	171	0.225 (0.147–0.346)	**<0.001**	0.702 (0.424–1.161)	0.168
Race	685				
Asian	13	Reference			
African American	33	1.578 (0.453–5.494)	0.473		
White	639	1.170 (0.374–3.657)	0.787		
COPZ2	698				
Low	348	Reference		Reference	
High	350	2.824 (2.190–3.642)	**<0.001**	1.210 (0.867–1.688)	0.263

WHO, world health organization; IDH, isocitrate dehydrogenase.

### COPZ2 co-expression network in glioma

**Figure 5:**
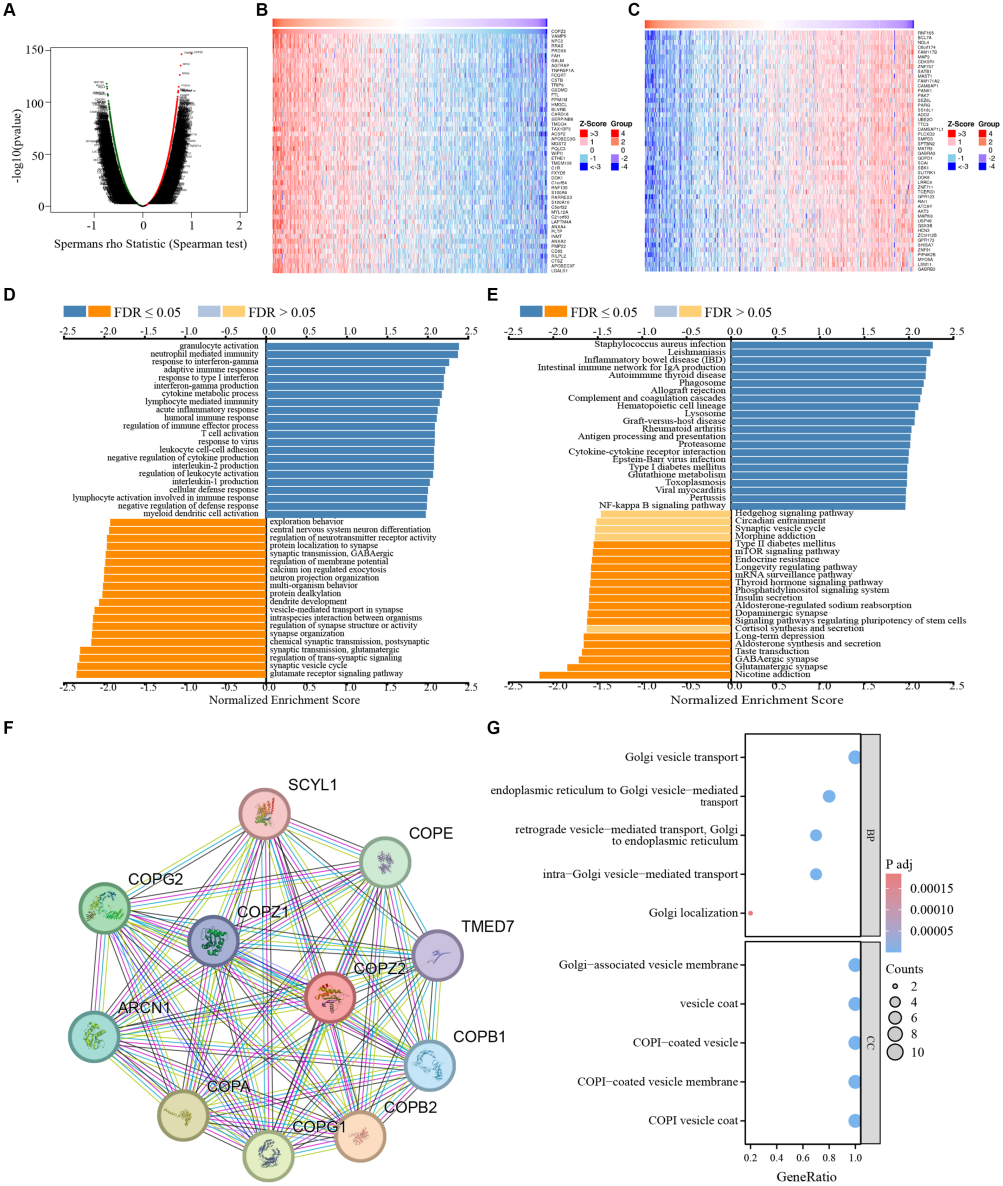
The COPZ2 co-expression genes in glioma. **(A)** Volcano map of co-expressed genes of COPZ2 in glioma cohort. Heatmap of the top 50 genes **(B)** positively and **(C)** negatively associated with COPZ2. **(D)** GO-BP and **(E)** KEGG pathway analysis of COPZ2 in glioma cohort. **(F)** Protein–protein interaction network obtained from STRING. **(G)** GO and KEGG pathway analysis of 10 COPZ2 -associated genes. BP, biological process.

To investigate the potential function and mechanism of COPZ2 in glioma, the Linkedomics database was applied to analyze the co-expressed networks of COPZ2 in glioma. As shown in Figure 5A, 2,447 genes were positively associated with COPZ2, and 1894 genes were negatively associated with COPZ2. The top 50 genes that were positively and negatively correlated with COPZ2 were displayed in Figures 5B,C. Next, Gene Ontology (GO) and Kyoto Encyclopedia of Genes and Genomes (KEGG) pathway analysis were performed to discover potential biological process (BP) terms and pathways. GO-BP analysis revealed that COPZ2 co-expressed genes mainly enriched in the neutrophil-mediated immunity, granulocyte activation, and response to interferon-gamma (Figure 5D). KEGG pathway analysis revealed that COPZ2 co-expressed genes mainly enriched in leishmaniasis, and staphylococcus aureus infection (Figure 5E). In addition, STRING database was used to construct the protein–protein interaction network of COPZ2 and its 10 co-expressed protein. The proteins interacted with COPZ2 were COPG1, COPG2, COPA, COPE, ARCN1, COPB1, COPB12, COPZ1, TMED7, and SCYL1 (Figure 5F). We then performed GO analysis of protein–protein interaction network of COPZ2, and showed that they were mainly enriched in Golgi−associated vesicle membrane, Golgi vesicle transport, and endoplasmic reticulum to Golgi vesicle−mediated transport (Figure 5G).

### Knockdown of COPZ2 suppresses the malignant behavior of glioma

**Figure 6:**
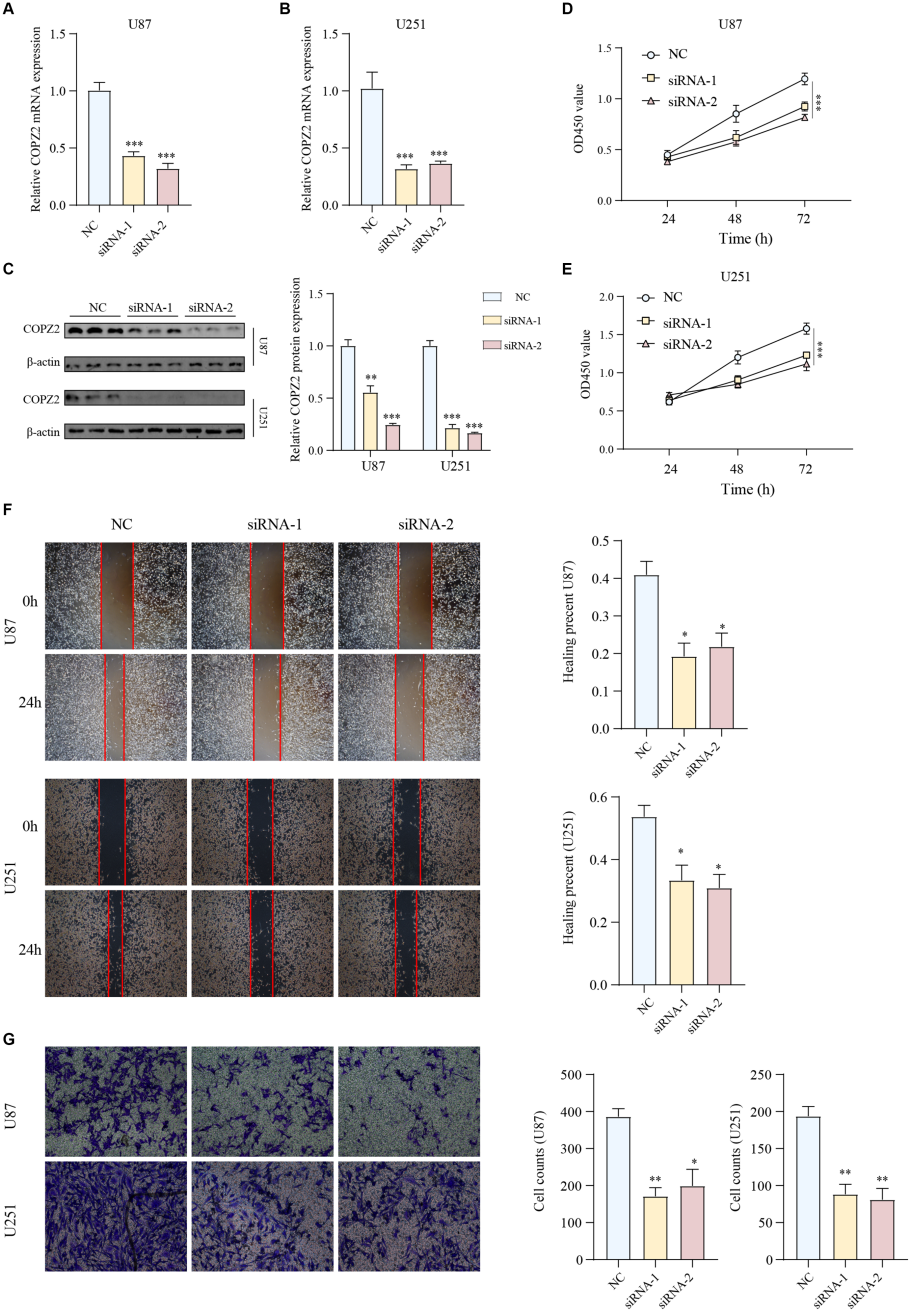
Knockdown of COPZ2 inhibited the malignant phenotypes of glioma cells. The knockdown efficiency of COPZ2 was detected in **(A)** U87 and **(B)** U251 cells. **(C)** The knockdown efficiency of COPZ2 in U87 and U251 glioma cells were detected by western blot. The proliferation capacity of **(D)** U87 and **(E)** U251 cells after COPZ2 knockdown at 24 h, 48 h, and 72 h. **(F)** The wound healing assay of the effect of COPZ2 knockdown on the U87 and U251 cell migration. **(G)** The transwell assay of the effect of COPZ2 knockdown on the U87 and U251 cell invasion. *p < 0.05, **p < 0.01, ***p < 0.001.

To further determine the biological functions of COPZ2 in the progression of glioma, siRNAs were used to silence endogenous COPZ2 expression in U87 and U251 glioma cell lines. U87 and U251 are the two most widely used glioma cell lines. U87 cells are of glial origin and express mutant PTEN, PI3K, and AKT. In addition, U251 cells are derived from astrocytes, and harbor PTEN mutation, upregulation of PI3K and AKT, and non-functional p53 (Schulz et al., 2022). The expression levels of the COPZ2 gene were quantified by qPCR and western blot (Figures 6A–C). As shown in Figures 6D,E, the CCK-8 assay revealed that the silencing of COPZ2 significantly suppressed the proliferation capacity of U87 and U251 cells. Wound healing results indicated that the migratory capacity of U87 and U251 cells was significantly inhibited at 24h after the knockdown of COPZ2 (Figure 6F). Similarly, transwell assays showed the invasion ability of U87 and U251 cells was decreased after COPZ2 knockdown (Figure 6G). These data indicated that COPZ2 was involved in the malignant behavior of glioma.

### Differentially expressed genes and functional enrichment analysis

**Figure 7:**
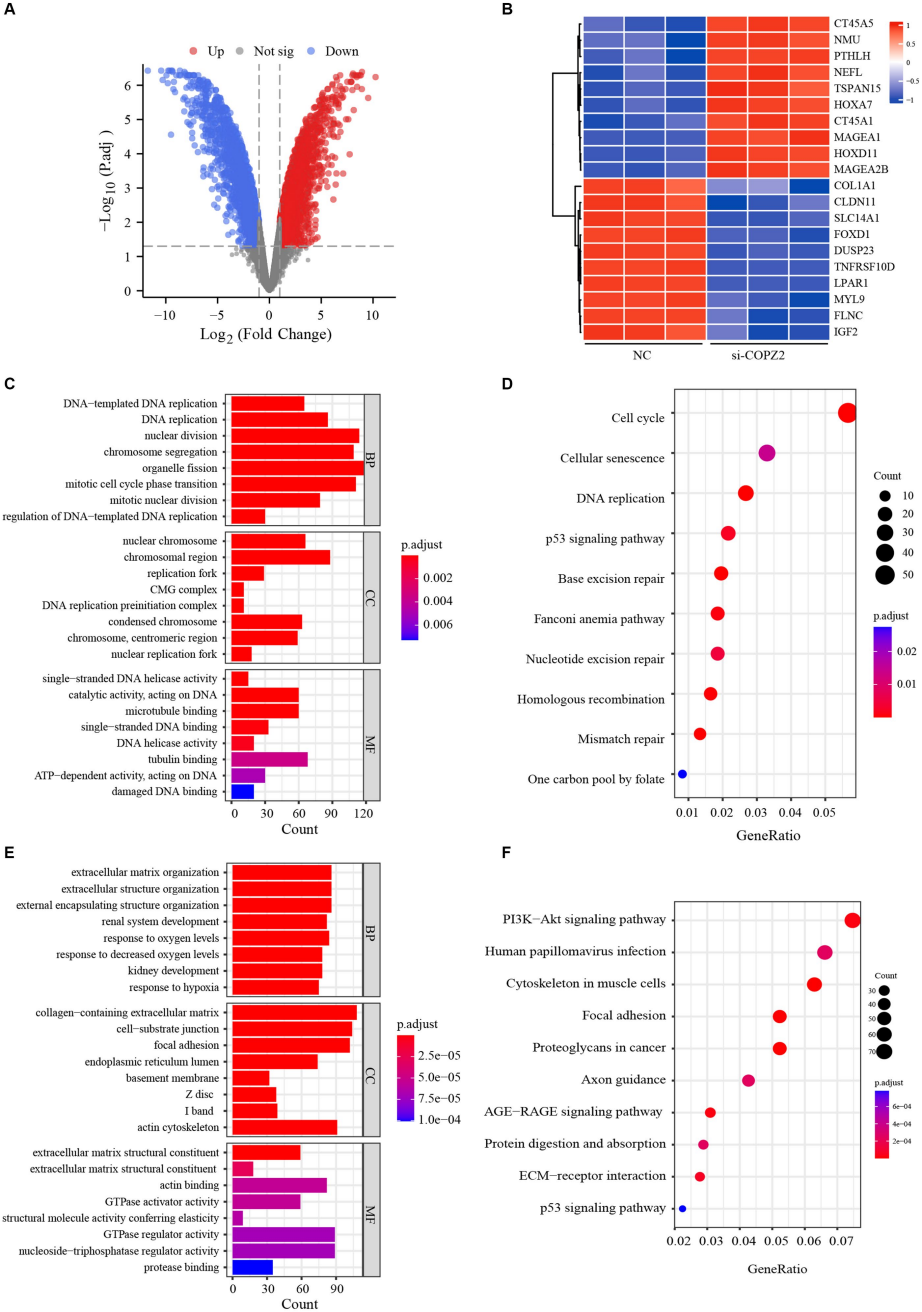
Gene expression profiles in U251 cells. **(A)** Volcano plot shows the differentially expressed genes between COPZ2-knockdown and control U251 cells. Red dots represent upregulated genes and blue dots represent downregulated genes. **(B)** Heat map of top 20 differentially expressed genes in U251 cells treated with or without siRNA. GO and KEGG analysis for **(C,D)** up-regulated genes and **(E,F)** down-regulated genes. CC, cellular component; BP, biological process; MF, molecular function.

**Figure 8:**
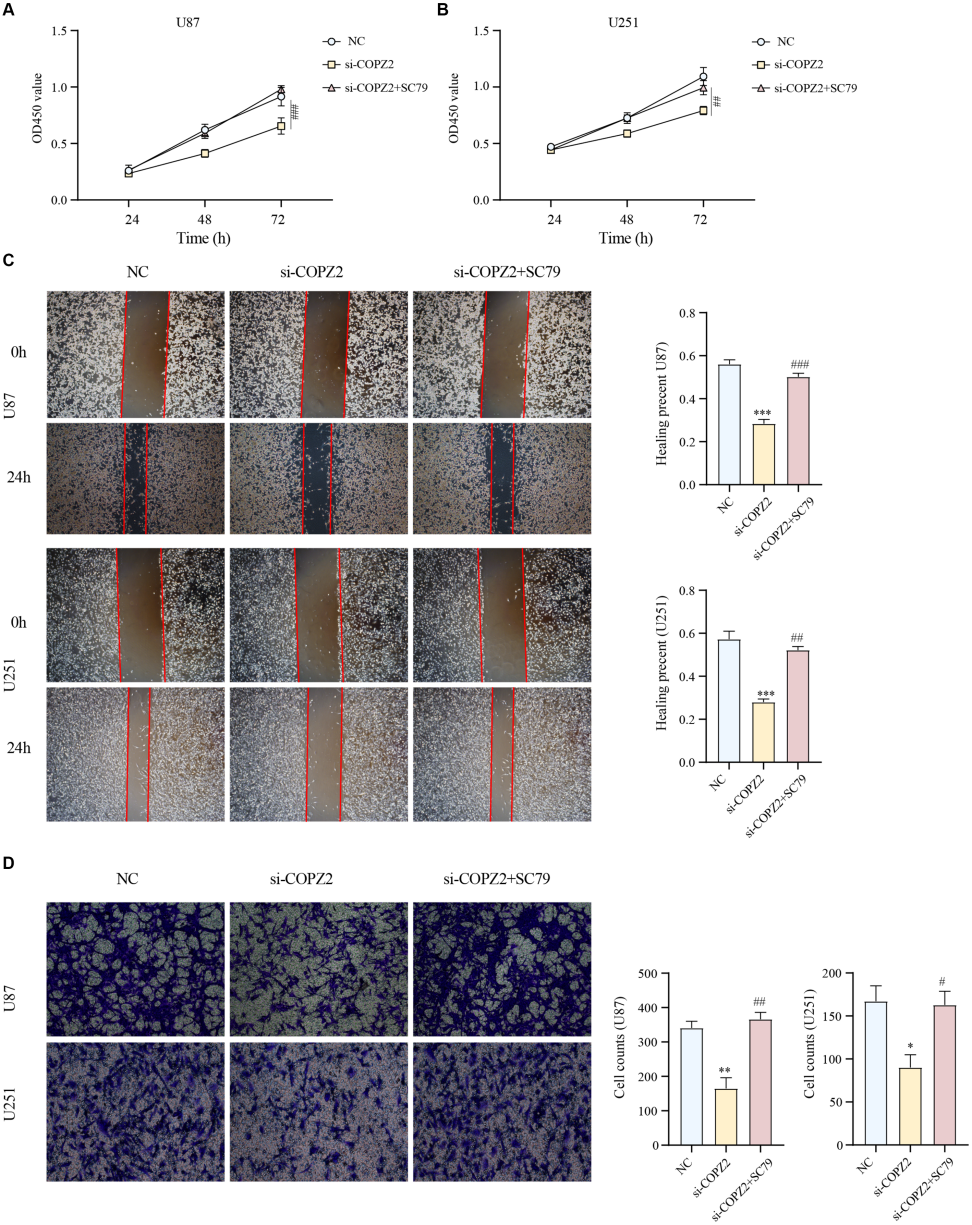
AKT activation reversed the antioncogenic effect of COPZ2 knockdown. The CCK-8 assay **(A,B)**, wound healing assay **(C)**, and transwell assay **(D)** of U87 and U251 cells after transfected with si-COPZ2 in the presence or absence of SC79 (5 μg/mL). *p < 0.05, **p < 0.01, ***p < 0.001 vs. NC group; #*p* < 0.05, ##*p* < 0.01, ###*p* < 0.001 vs. si-COPZ2 group.

To identify the potential mechanism underlying COPZ2-driven malignant behaviors in glioma, we performed a microarray assay using the COPZ2-knockdown and control U251 cell lines. A total of 4,846 differentially expressed genes in COPZ2-knockdown cells were identified, including 2,486 upregulated genes and 2,360 downregulated genes (Figure 7A). The top 20 differentially expressed genes were presented in Heatmap (Figure 7B). The top 10 upregulated genes included CT45A5, NMU, PTHLH, NEFL, TSPAN15, HOXA7, CT45A, MAGEA1, HOXD11, and MAGEA2B. The top 10 downregulated genes included IGF2, FLNC, MYL9, LPAR1, TNFRSF10, DDUSP23, FOXD1, SLC14A1, CLDN11, and COL1A1. GO analysis revealed that upregulated genes mainly participated in DNA − templated DNA replication, nuclear chromosome, and single−stranded DNA helicase activity (Figure 7C). KEGG analysis revealed that upregulated genes were mainly enriched in the cell cycle, cellular senescence, and DNA replication (Figure 7D). In addition, the downregulated genes of GO analysis were enriched in the extracellular matrix organization, collagen−containing extracellular matrix, extracellular matrix structural constituent (Figure 7E). The downregulated genes of KEGG analysis were enriched in PI3K − AKT signaling pathway (Figure 7F). These results indicated that COPZ2-knockdown may exert its antitumor effect by inhibiting the activity of the PI3K/AKT signaling pathway. Thus, to test whether COPZ2-driven malignant behaviors in glioma cells through PI3K − AKT signaling pathways, rescue experiments were conducted by treating U251 and U87 cells with SC79 (PI3K − AKT signaling pathways activator). The results indicated that SC79 treatment significantly reversed the inhibitory effects of COPZ2-knockdown on malignant behaviors (Figure 8).

## Discussion

Glioma is a common tumor of the central nervous system, and its occurrence and development may involve multiple factors. Firstly, genetic factors are closely related to the occurrence of glioma. Studies have shown mutations in certain genes, such as TP53, which play a critical role in tumor suppression (Olivier et al., 2010). Secondly, environmental factors, including long-term exposure to electromagnetic radiation, chemicals, or radiation, can also increase the risk of glioma. Thirdly, abnormalities in the immune system may contribute to glioma development, affecting the body’s ability to detect and destroy abnormal cells. Lastly, cell signaling pathways, such as the epidermal growth factor receptor (EGFR) (Sabbah et al., 2020), PI3K-AKT (Xia and Xu, 2015), and RAS-MAPK (Masliah-Planchon et al., 2016), are significantly involved in the progression of glioma, influencing cell growth, survival, and proliferation. In conclusion, the occurrence and development of glioma is a complex process influenced by multiple factors.

The findings of this study reveal a significant association between the expression level of COPZ2 and the disease status of glioma. Specifically, COPZ2 expression correlates positively with WHO grading, indicating higher levels in more advanced grades of the tumor. Additionally, patients with IDH mutation or 1p/19q co-deletion exhibit lower COPZ2 levels, suggesting a potential inverse relationship between these genetic alterations and COPZ2 expression. Notably, Kaplan–Meier survival analysis indicates that elevated COPZ2 expression serves as an independent predictor of overall survival in glioma patients, with higher COPZ2 levels associated with poorer prognosis. Moreover, results from univariate and multivariate Cox regression analyses demonstrate that COPZ2 is a significant prognostic factor for unfavorable outcomes. Forest plots and calibration plots further validate these findings, while ROC curves highlight the predictive accuracy of COPZ2 as a prognostic marker. The robustness of these analyses underscores the potential clinical utility of COPZ2 in stratifying glioma patients based on risk and tailoring treatment strategies accordingly. In essence, the clinical data analysis underscores the potential of COPZ2 as a valuable prognostic biomarker for glioma. Its association with disease severity and patient survival suggests that COPZ2 could be targeted in the development of novel treatment modalities, potentially improving outcomes for glioma patients. This study highlights the importance of further research into the molecular mechanisms underlying COPZ2’s role in glioma to fully harness its prognostic and therapeutic potential.

To further comprehend the role of COPZ2 in glioma, gene–gene interaction (GGI) and protein–protein interaction (PPI) networks were established. The data illustrate that COPZ2 primarily participates in Golgi vesicle transport-related functions and pathways. In glioma, dysregulation of COPZ2 disrupts normal vesicular trafficking and protein sorting (Feng et al., 2021). This can lead to the mislocalization of key signaling molecules and receptors, thereby altering cell signaling pathways that promote tumorigenesis. It is also due to the obstruction of vesicle transport that naturally damages various vesicle transport problems in autophagy flow. COPZ2 has been implicated in the regulation of autophagy (Shtutman et al., 2011). In glioma cells, overexpression of COPZ2 might enhance autophagic flux, thereby providing a survival advantage under stress conditions such as hypoxia and nutrient deprivation, common in the tumor microenvironment. Future investigations into the mechanisms of COPZ2 in glioma should also consider the interplay and changes between autophagy regulation and vesicle transport. Notably, the PI3K/AKT pathway, which is often hyperactivated in glioma, can be influenced by COPZ2-mediated vesicular trafficking (Giussani et al., 2009; Langhans et al., 2017). COPZ2 dysregulation may lead to aberrant localization and activation of key components of this pathway, thereby enhancing cell proliferation and survival. Additionally, co-expression analysis and functional enrichment analysis indicate that genes co-expressed with COPZ2 in glioma are predominantly associated with neutrophil-mediated immunity, granulocyte activation, and response to interferon-gamma. In the context of gliomas, dysregulation of vesicular trafficking and protein sorting can lead to the mislocalization of key signaling molecules (Vascotto et al., 2007), disrupting normal cellular functions and promoting tumorigenesis.

To further validate the role of COPZ2 in glioma, siRNA-mediated knockdown of COPZ2 was performed in two glioma cell lines. The outcomes revealed that COPZ2 knockdown impeded the proliferation, migration, and invasion capabilities of glioma cells. Subsequent microarray analysis uncovered differential expression of numerous cancer-related genes and impacted diverse cancer-associated pathways following COPZ2 knockdown. Then, the antitumor effect of COPZ2 knockdown was reversed by an activator of the PI3K/AKT signaling pathway. Thus, COPZ2 emerges as a pivotal pro-oncogenic factor in the progression of glioma. In addition, to confirm the exact mechanism role of COPZ2 in gliomas, the sequencing and glioma malignant behavior results of the above studies should be further validated in multiple cell lines and animal experiments, which will be further explored in our future investigation.

In conclusion, our research findings suggested that COPZ2 was highly expressed and associated with poor prognosis. Furthermore, the knockdown of COPZ2 significantly inhibited the proliferation, migration, and invasion abilities of U251 and U87 cells via inhibition of the PI3K/AKT signaling pathway, indicating that COPZ2 could promote the progression of glioma. Given these revelatory insights, COPZ2 could potentially serve as a novel diagnostic or therapeutic target for glioma.

## Data availability statement

The raw data supporting the conclusions of this article will be made available by the authors, without undue reservation.

## Author contributions

ZG: Writing – original draft, Writing – review & editing, Conceptualization, Data curation, Formal analysis, Methodology, Software. CM: Writing – review & editing, Data curation, Formal analysis, Methodology. YQ: Writing – review & editing, Data curation, Formal analysis, Methodology. YT: Writing – review & editing, Data curation, Formal analysis, Methodology. MS: Investigation, Validation, Writing – review & editing. CT: Writing – original draft, Writing – review & editing, Conceptualization, Data curation, Formal analysis, Methodology, Software, Supervision, Validation, Visualization. LZ: Writing – original draft, Writing – review & editing, Conceptualization, Data curation, Formal analysis, Methodology, Software, Supervision, Validation, Visualization.

## Footnotes

^1^https://portal.gdc.cancer.gov/

^2^http://timer.cistrome.org/

^3^http://ualcan.path.uab.edu/

^4^http://www.linkedomics.org/login.php

^5^https://cn.string-db.org/
